# Acute necrotic pancreatitis after esophagectomy: a case report

**DOI:** 10.1186/s40792-015-0033-5

**Published:** 2015-03-26

**Authors:** Keisuke Kawamorita, Yasuhiro Tsubosa, Yurika Oka, Satoru Matsuda, Katsushi Takebayashi, Masahiro Niihara, Yukiyasu Okamura, Katsuhiko Uesaka

**Affiliations:** Division of Esophageal Surgery, Shizuoka Cancer Center Hospital, 1007 Shimonagakubo, Nagaizumi-cho, Sunto-gun, Shizuoka 411-8777 Japan; Division of Hepato-Biliary-Pancreatic Surgery, Shizuoka Cancer Center Hospital, 1007 Shimonagakubo, Nagaizumi-cho, Sunto-gun, Shizuoka 411-8777 Japan

**Keywords:** Esophageal carcinoma, Postoperative complication, Acute pancreatitis, Esophagectomy, Gastric tube necrosis

## Abstract

Acute pancreatitis after esophagectomy is a very rare but fatal complication. This case report describes a 74-year-old man diagnosed with cT2N0M0, cStage IB esophageal squamous cell carcinoma (Union for International Cancer Control, seventh edition). On the basis of the patient’s condition, it was decided that he should undergo esophagectomy without thoracotomy. The patient developed pyrexia 3 days after the operation. Chest and abdominal computed tomography revealed severe acute pancreatitis and gastric tube necrosis; therefore, gastrectomy was performed. Subsequent surgical exploration indicated pancreatic necrosis that was diagnosed as acute necrotic pancreatitis. Postoperative management of acute pancreatitis and the general condition of the patient were quite challenging, and rapid deterioration of the respiratory status was observed. The patient experienced multiple organ failure and died 57 days after the second surgery (60 days after the first surgery). This is a report of a patient with acute necrotic pancreatitis after esophagectomy.

## Background

The rate of complications after esophagectomy is high, at 45% to 58.4% [1,2]. Respiratory complications are the most common, while the incidence of postoperative acute pancreatitis is low, at approximately 0.4% to 0.5% [3].

When acute pancreatitis develops due to a synergistic effect with the condition of the patient after an esophagectomy, the pathology becomes complicated, leading to a requirement for long-term multidisciplinary care.

Herein, we report a case of acute necrotic pancreatitis after esophagectomy.

## Case presentation

The patient was a 74-year-old man who was referred to our hospital after an upper endoscopy during a medical check-up revealed a flat, elevated lesion located in the lower thoracic esophagus. Histologically, this was diagnosed as squamous cell carcinoma. Specifically, the lesion was cT2N0M0, cStage IB esophageal squamous cell carcinoma (Union for International Cancer Control, seventh edition). The patient also had two early gastric cancer lesions of the lesser curvature of the stomach. In hematological examination, mild elevation of serum creatinine was observed, on the other hand, there were no other abnormal values including serum amylase, aminotransferases, C reactive protein (CRP). Body mass index (BMI) was calculated to be 22.2 kg/m^2^.

Therefore, this patient had interstitial pneumonia and severe obstructive respiratory disease (forced expiratory volume 1.0(s)% (FEV1.0%): 44.6%), esophagectomy with thoracotomy was considered accompanying greater risks. Consequently, the patient underwent transhiatal esophagectomy, without thoracotomy. Gastric tube reconstruction was performed via a postmediastinal route. Lymph nodes along the left gastric artery located in superior border of the pancreas were dissected with gentle traction of the pancreas. There were no metastatic lymph nodes in surgical findings.

**Figure 1:**
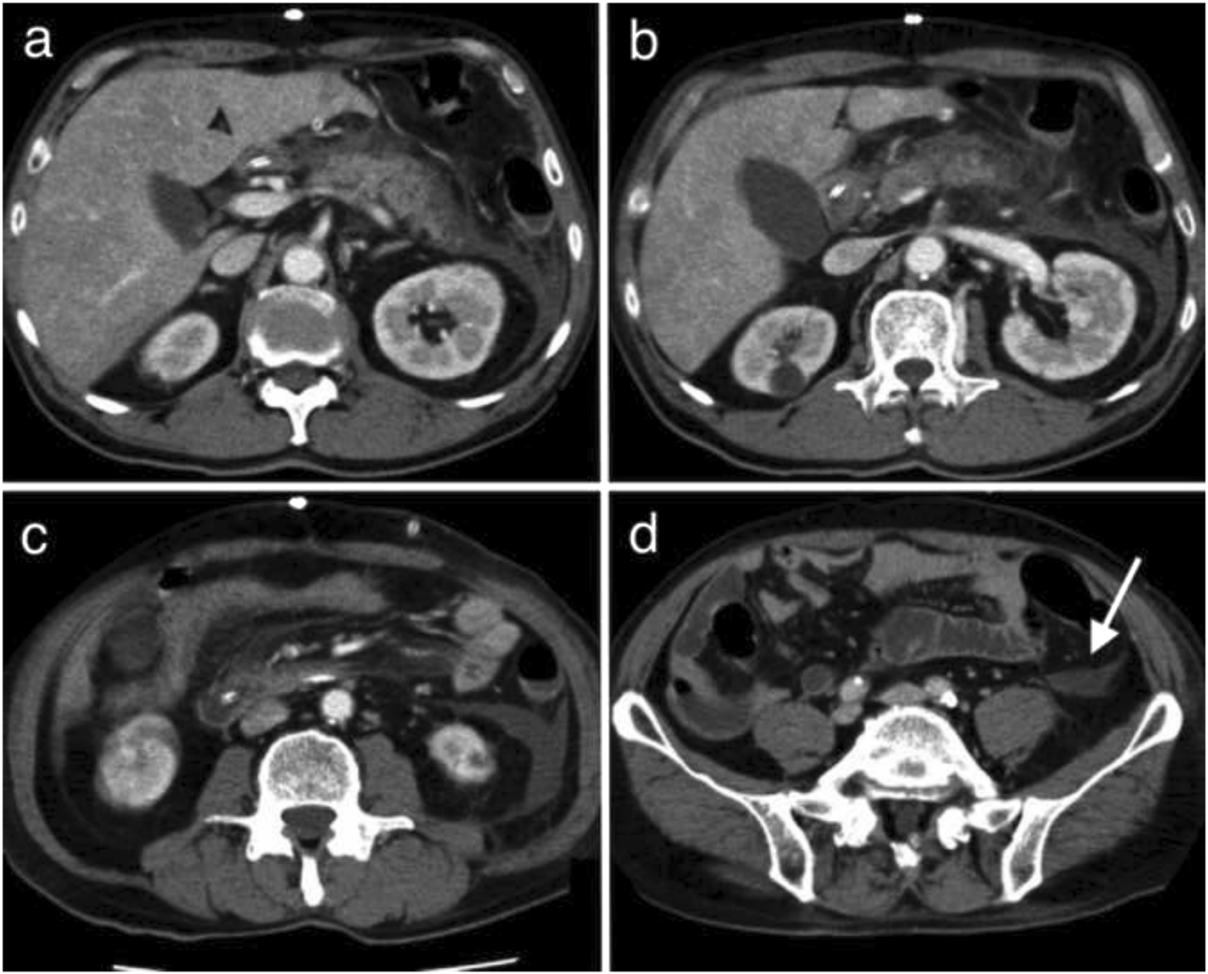
**Abdominal contrast computed tomography findings.** Increased density of adipose tissue surrounding the pancreas and some areas without contrast enhancement in the pancreatic body was observed **(a**-**c)**. Furthermore, inflammation extended from the inferior pole of the left kidney to the pelvic region **(d)**. On the basis of the findings on the computed tomography scans, the patient was diagnosed with severe acute grade 3 pancreatitis (Japanese Guidelines for diagnosis of acute pancreatitis 2010, third version).

**Figure 2:**
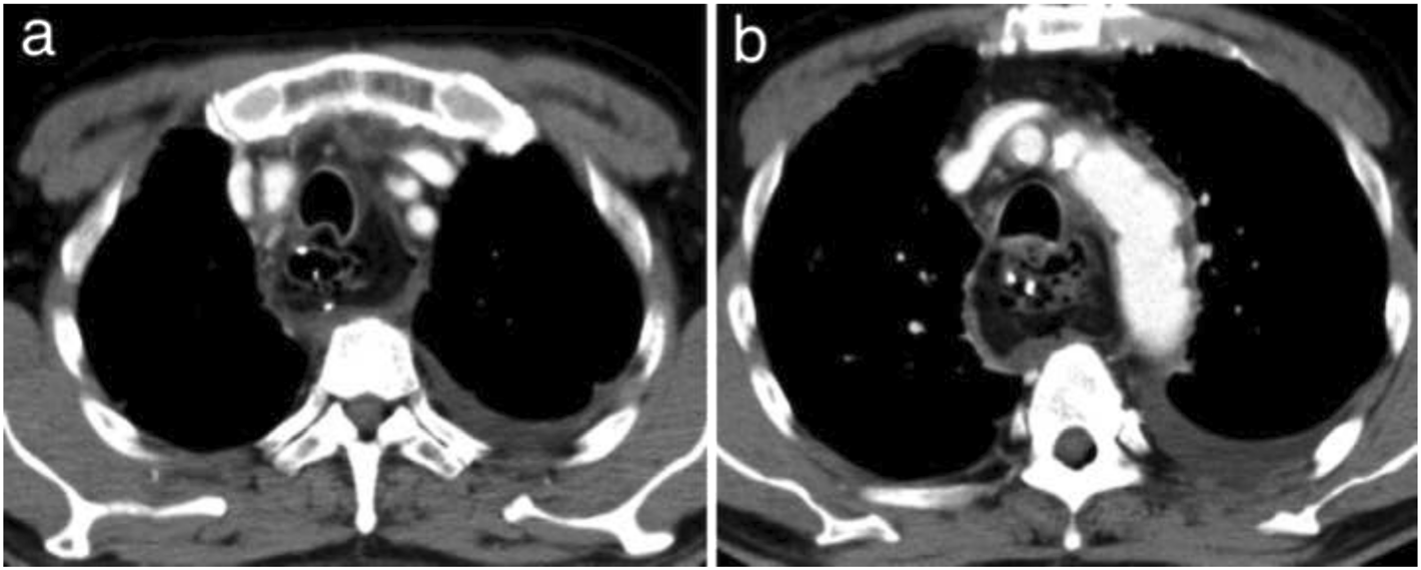
**Chest contrast computed tomography findings.** Contrast enhancement was significantly less in the wall at the fundus of the stomach and the vessels in the greater omentum **(a)**. Pneumatosis intestinalis was observed in the gastric wall. According to the findings, this was diagnosed as gastric tube necrosis **(b)**.

**Figure 3:**
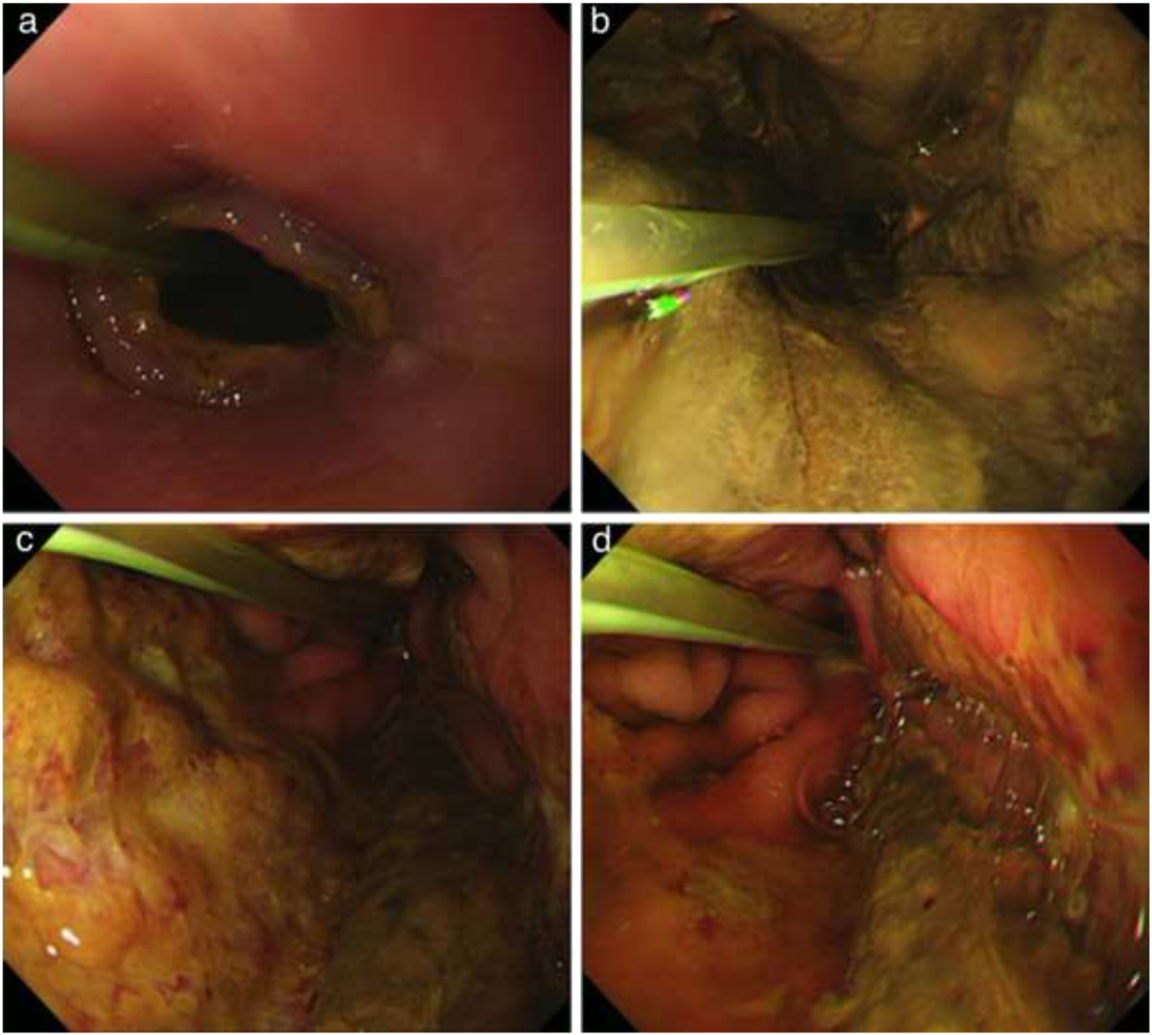
**Upper endoscopy findings.** Necrosis of the membrane spread from the fundus to the center of the gastric tube **(a**-**d)**.

Three days after surgery, the patient developed vomiting and pyrexia. Aspiration pneumonia was suspected; therefore, chest and abdominal computed tomography (CT) were performed, and they revealed increased density of the adipose tissue surrounding the pancreas and an accumulation of exudate in the retroperitoneum beyond the inferior pole of the left kidney (Figure 1). An upper endoscopy was also performed because of the decreased contrast enhancement, and the presence of pneumatosis intestinalis at the fundus of the stomach was detected (Figure 2). This finding confirmed extensive gastric tube necrosis (Figure 3). On the basis of these findings, the patient was diagnosed with severe acute pancreatitis and gastric tube necrosis secondary to acute pancreatitis. The patient underwent emergency total gastrectomy, cervical esophagostomy, and jejunostomy to allow enteral feeding. The patient’s serum amylase level was high (2,604 U/L) the day after the initial surgery, but this level rapidly decreased to 137 U/L on the day acute pancreatitis was diagnosed.

**Figure 4:**
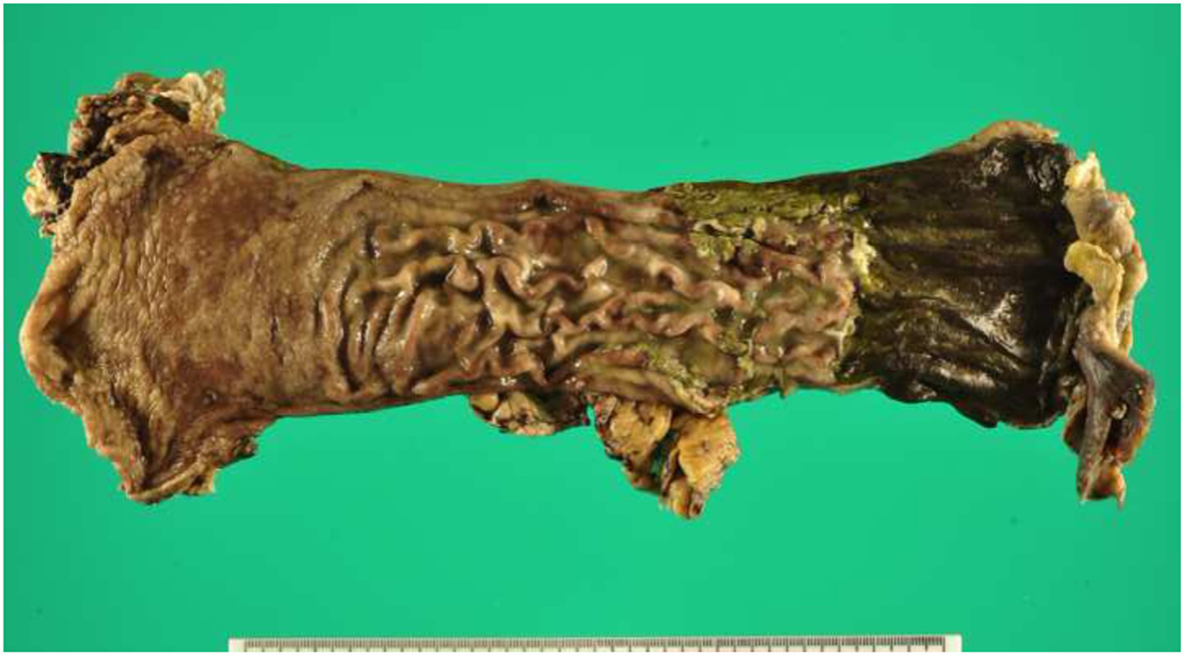
**Resected specimen findings.** Mucosal necrosis was observed extending 60 mm from the edge of the gastric tube.

The operation revealed the presence of saponification on the pancreatic body and in the greater omentum along the antrum of the stomach, as well as severe inflammation extending from the pancreatic body to the pancreatic head. Furthermore, the saponification also revealed from the retroperitoneum to the mesentery proper. Gastric necrosis extended to approximately 60 mm from the anastomotic site (Figure 4).

After the second surgery, an antimicrobial agent and gabexate mesilate were administered to treat the acute pancreatitis. Contrast CT was performed again, and abscesses were drained via CT-guided puncture of abscess cavities. Although the abscess cavities around the pancreas gradually decreased, necrotic tissue lysis persisted. There was also a possible evidence of pancreatitis in the retroperitoneum, from the pancreas to the left pelvic cavity. Puncture was attempted, but effective drainage was not achieved (Figure 1d, arrow line).

It was quite difficult to provide the patient with adequate nutrition. The patient received the best possible nutritional therapy, including the feeding method, drug administration, and optimized nutrient composition, but his nutrition status did not improve.

The patient’s respiratory status progressively deteriorated during his prolonged hospitalization period, and he developed adult respiratory distress syndrome (ARDS) due to aspiration. Thereafter, the patient’s condition rapidly progressed to multiple organ failure, and he died 57 days after the second surgery (60 days after the first surgery). The autopsy was not performed.

## Discussion

The incidence rate of acute pancreatitis as a complication after esophagectomy is very low, occurring in only approximately 0.4% to 0.5% of all patients who undergo esophagectomies [3,4]. There have been some cases of acute pancreatitis secondary to ileus during the long-term follow-up of esophagectomy patients [5], but there has only been one other report in the entire world, besides ours, of acute pancreatitis immediately after surgery [3].

Blom et al. reported four patients with acute pancreatitis after they underwent esophagectomies; two of the four patients died [3]. For all the reported cases, including our patient, three out of five patients died owing to acute pancreatitis after esophagectomy. This suggests that the fatality rate of acute pancreatitis after surgery for esophageal cancer is quite high (Table 1).

**Table 1:** **Cases of postoperative acute pancreatitis**

**Case**	**Author [ref]**	**Year**	**Age/sex**	**Background**	**Operation**	**BMI**	**Presentation of pancreatitis**	**Amylase level on the day of diagnosis (U/l)**	**Diagnosis**	**Necrosis of gastric tube**	**Prognosis**	**Cause of death**
1	Blom et al. [3]	2009	73/M	-	TTE	24	2 POD	1,690	Relaparotomy	+	Dead, 22 POD	MOF
2	Blom et al. [3]	2009	72/M	Alcohol abuse	TTE	25	12 POD	338	Relaparotomy	-	Alive	-
3	Blom et al. [3]	2009	52/M	MI	THE	32	6 POD	110	Autopsy	-	Dead, 6 POD	Electromechanical dissociation
4	Blom et al. [3]	2009	78/M	-	THE	26	12 POD	108	Relaparotomy	+	Alive	-
5	Our case	2014	74/M	IP, COPD, alcohol abuse	THE	23	3 POD	137 (maximum: 2,604)	CT	+	Dead, 60 POD	MOF

M*,* male; F, female; MI, myocardial infarction; IP, interstitial pneumonia; COPD, chronic obstructive pulmonary disease; TTE transthoracic esophagectomy; THE, transhiatal esophagectomy; BMI, body mass index; POD, post operative day; MOF, multiple organ failure.

A diagnosis of postoperative acute pancreatitis is based on not only the physical symptoms, such as upper abdominal pain, but also the serum amylase level, serum lipase level, and CT images [3,6], similar to the procedure for diagnosis of acute pancreatitis unrelated to surgery. In general, physical symptoms are nonspecific and difficult to differentiate from postoperative wound pain. Therefore, this information is insufficient for diagnosis. Furthermore, the positive predictive value of serum amylase levels is 15% to 72%, which is not high enough to diagnose acute pancreatitis [7]. Serum amylase levels decrease more rapidly than serum lipase levels, suggesting that serum amylase levels may not be an appropriate index for diagnosis, depending on the timing of the test [8]. On the other hand, the positive predictive value of the serum lipase level is 90% [7]; therefore, it is a more accurate index of postoperative pancreatitis than the serum amylase level. CT scans cannot always be used to differentiate acute pancreatitis from pancreatitis after esophagectomy. Therefore, postoperative acute pancreatitis is difficult to diagnose and is sometimes overlooked [3]. In fact, one of the four patients with acute pancreatitis after esophagectomy in the report by Blom et al. was diagnosed with acute pancreatitis at autopsy [3].

The cause of the acute pancreatitis after the esophagectomy could not be identified for the patient in this case report. The patient had a history of heavy alcohol use, but no high-risk findings of pancreatitis, such as gallstones, were observed on the preoperative CT images. Suggested etiological factors for acute pancreatitis include intraoperative damage to the pancreatic parenchyma, mobilization of the duodenum, or compromised vascularization in the pancreatic area either by ischemic injury due to hypovolemic shock or by a thromboembolism [6,9-12]. However, none of these factors were identified as the cause of pancreatitis in our patient. Furthermore, there was a report that exclusion and traction of the pancreas during dissection around the celiac artery may stimulate the organ or cause obstruction of the main pancreatic duct [3]. All the previously mentioned five cases, including our case, had high body mass indexes; therefore, stronger-than-usual exclusion force may have been applied to the pancreas in order to visualize intra-abdominal manipulation.

Kuo et al. and Lubianskii et al. suggested the possibility that contraction at the Vater papilla during the perioperative period might induce acute pancreatitis [6,13]. Fentanyl was administered to our patient through an epidural catheter to control perioperative pain. Fentanyl causes constriction of the sphincter of Oddi, but the contraction caused is weaker than that caused by morphine [14]. As fentanyl, which is administered into the epidural space, is transferred from the plexus venous in the epidural space to the blood [15], the effect of fentanyl on the sphincter of Oddi may promote spasms of the Vater papilla. This may damage the mechanism by which pancreatic juices are excreted, thereby inducing the onset of acute pancreatitis.

The patient in our study had to undergo a total gastrectomy owing to extensive gastric tube necrosis. The findings at the time of the second surgery showed the presence of saponification on the pancreatic body as well as inflammation extending from the pancreatic body, to the pancreatic head, to the greater omentum, which might cause severe inflammation in perfusion area of the right gastroepiploic artery and vein, leading to gastric circulatory failure and extensive necrosis. As described above, Blom et al. reported four patients with acute pancreatitis; two of these four patients had gastric tube necrosis. Therefore, it is probable that gastric tube necrosis occurs subsequently to acute pancreatitis after esophagectomy. Remarkable necrosis arising the tip of gastric tube can be considered that indicating severe circulatory failure of the whole gastric tube caused by problems with main vessels which perfuse gastric tube. Thus, this case found this finding, it is necessary to examine carefully about abdominal cavity using CT, especially the area of right gastric artery or vein and right gastroepiploic artery or vein which mainly perfuse gastric tube, considering to acute pancreatitis.

Acute pancreatitis is sometimes associated with aneurysm formation and rupture in the splenic artery and other arteries [8]. Exposed arteries or arterial resection margins during lymph node dissection for esophagectomies may increase the risk of aneurysm formation. It is important to observe characteristics of the drainage and aneurysm formation, which are visible on contrast CT images (three-dimensional images of the arteries).

## Conclusions

Acute pancreatitis after esophagectomy is a very rare but difficult-to-treat and often fatal complication that requires long-term multidisciplinary care. In the case of gastric necrosis after esophagestomy, it should be examined considering acute pancreatitis.

## Consent

The informed consent was obtained from the kin of the patient for the publication of this report and any accompanying images.
